# Characterization of the complete mitochondrial DNA sequence of the *Lagocephalus guentheri* (Tetraodontidae, Tetraodontiformes)

**DOI:** 10.1080/23802359.2020.1823278

**Published:** 2020-10-05

**Authors:** Xuanyun Huang, Yongfu Shi, Dongmei Huang, Xiaosheng Shen, Yuan Wang, Jiajie Chen, Youqiong Cai

**Affiliations:** Key Laboratory of East China Sea Fishery Resources Exploitation, Ministry of Agriculture and Rural Affairs, East China Sea Fisheries Research Institute, Chinese Academy of Fishery Sciences, Shanghai, China

**Keywords:** Complete mitochondria genome, *Lagocephalus guentheri*, Tetraodontidae, phylogenetic analysis

## Abstract

The complete mitochondrial genome of *Lagocephalus guentheri* was reported in the present study, which was 16,461 bp in length. It consists of 13 protein-coding genes, two ribosomal RNA genes, 22 transfer RNA genes and a non-coding control region. The overall base composition of the genome is 27.54% for A, 24.80% for T, 31.23% for C and 16.43% for G. The phylogenetic tree, which is based on 12 protein-coding gene sequences, suggested that *L. guentheri* was closest to *L. spadiceus*. This study could give impetus to studies focused on population structure and molecular evolution of *L. guentheri*.

*Lagocephalus guentheri* belongs to the family Tetraodontidae, subfamily Tetraodontinae. This species is distributed in the Indo-West Pacific, including Persian Gulf, southern Oman, India, Indonesia, northwestern Australia and the South China Sea (Randall 1995). The complete mitochondrial genome of *L. guentheri* was reported and characterized herein, which provides significant information for further studies on its taxonomy and population genetics.

The sample of *L. guentheri* was collected from Bohai Bay. DNA materials were extracted from muscle tissues using the TIANamp Marine Animals DNA Kit (TIANGEN Biotech, Beijing, China). The complete mitochondrial genome was obtained through de novo assembly of 1.86 Gb Illumina Hiseq data in PE150 mode.

The mitochondrial genome DNA of *L. guentheri* was a circular molecular of 16,461 bp in length with a predicted control region of 835 bp in length. This genome contains 13 protein-coding genes (PCGs), 22 transfer RNA (tRNAs), and two ribosomal RNA (rRNAs). Two kinds of start codons (ATG and GTG) were identified in 13 protein-coding genes; nine genes ended with TAA, two genes ended with AGA, one gene ended with TAG, whereas the other gene had incomplete stop codons T—. Among the 37 genes, 28 were encoded by heavy strand, while nine were encoded by light strand just as in other teleosts. The gene arrangement of the mitogenome was the same with other species in genus *Lagocephalus* (Jiang et al. 2018; Xu et al. 2019). The nucleotide composition was 27.54% for A, 24.80% for T, 31.23% for C and 16.43% for G.

To examine the phylogenetic status of *L. guentheri*, nucleotide sequences of 12 protein-coding genes except for ND6 of 13 Tetraodontidae species and one *Benthosema pterotum* as the outgroup were aligned by the MAFFT version 7 software (Katoh and Standley 2013). Phylogenetic analysis was conducted based on maximum likelihood (ML) analyses implemented in IQ-TREE 1.5.5 (Nguyen et al. 2015) under the default model. Support for the inferred ML tree was inferred by bootstrapping with 1000 replicates (Figure 1). It revealed that *L. guentheri* had the closest relationship with *L. spadiceus*. Species in genus *Lagocephalus* were clustered into one clade.

**Figure 1.**
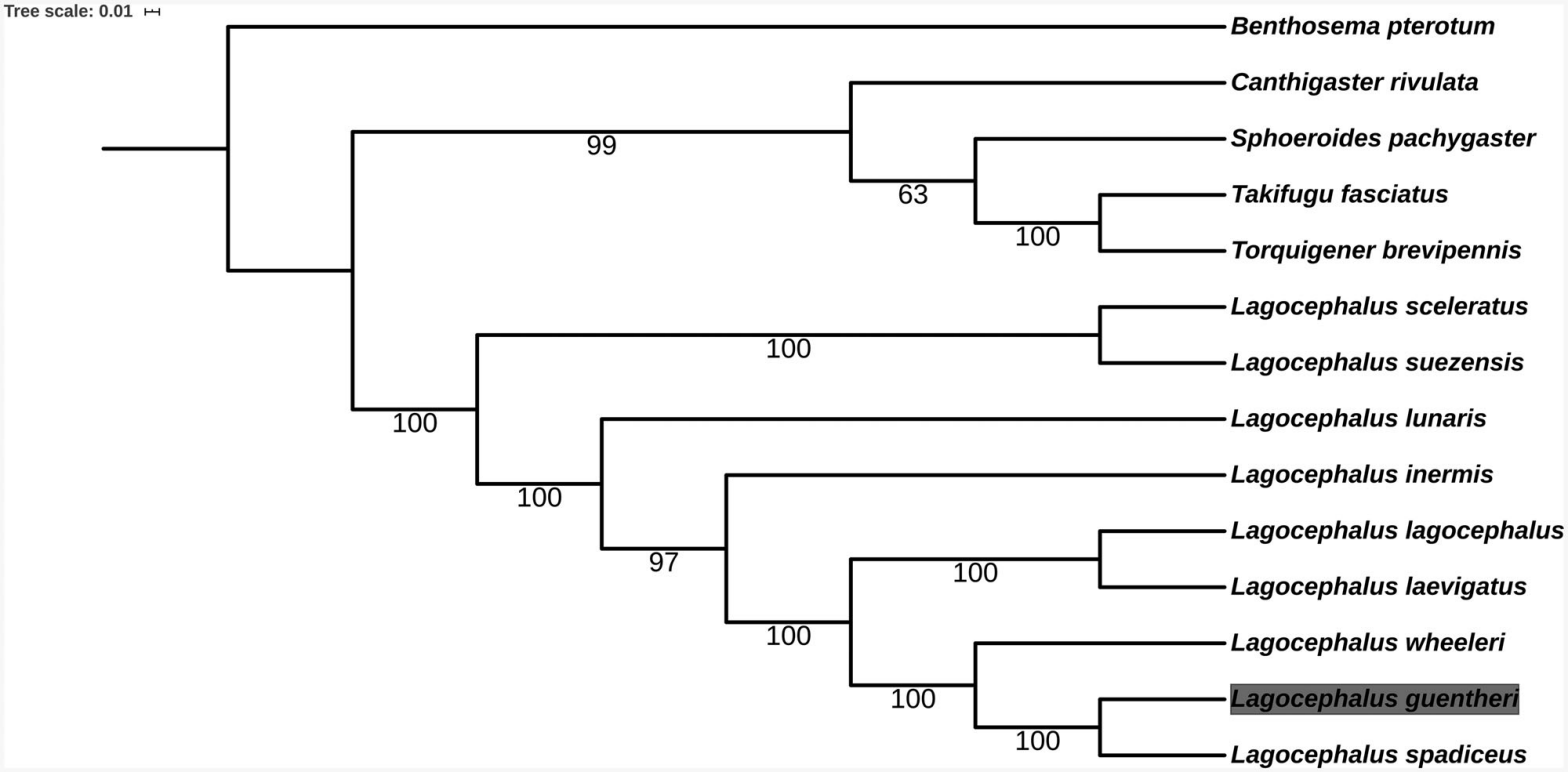
Phylogenetic relationships among 13 Tetraodontidae species and one *Benthosema pterotum* as the outgroup based on 12 PCGs. Bootstrap support values are given at the nodes. Mitochondrial genome accession number used in this phylogeny analysis: *L*agocephalus *sceleratus* MH550879.1; *L. lunaris* GQ461750.1; *L. lagocephalus* AP011933.1; *L. spadiceus* NC_026194.2; *L. suezensis* NC_026229.1; *L. inermis* NC_029376.1; *L. wheeleri* AP009538.1; *L. laevigatus* NC_015345.1; *Takifugu fasciatus* NC_032400.1; *Sphoeroides pachygaster* AP006745.1; *Torquigener brevipennis* AP009537.1; *Canthigaster rivulata* AP006744.1; *Benthosema pterotum* NC_047480.1.

## Data Availability

The data that support the findings of this study are openly available in GenBank of NCBI at https://www.ncbi.nlm.nih.gov, reference number MT903227.
